# Asymmetrical Forces Dictate the Distribution and Morphology of Platelets in Blood Clots

**DOI:** 10.3390/cells10030584

**Published:** 2021-03-06

**Authors:** Tatiana A. Kovalenko, Marie-Noelle Giraud, Anita Eckly, Anne-Sophie Ribba, Fabienne Proamer, Sandrine Fraboulet, Nadezhda A. Podoplelova, Jeremy Valentin, Mikhail A. Panteleev, Carmen Gonelle-Gispert, Stéphane Cook, Laurence Lafanechère, Anastasia N. Sveshnikova, Karin Sadoul

**Affiliations:** 1 Center for Theoretical Problems of Physico-Chemical Pharmacology, Russian Academy of Sciences, 30 Srednyaya Kalitnikovskaya str., 109029 Moscow, Russia; after-ten@yandex.ru (T.A.K.); podoplelovaN@yandex.ru (N.A.P.); mapanteleev@yandex.ru (M.A.P.); 2 Cardiology, Faculty of Science and Medicine, University of Fribourg, CH-1700 Fribourg, Switzerland; marie-noelle.giraud@unifr.ch (M.-N.G.); jeremy.valentin@extern.insel.ch (J.V.); stephane.cook@unifr.ch (S.C.); 3 BPPS UMR-S 1255, EFS Grand Est, FMTS, INSERM, University of Strasbourg, F-67065 Strasbourg, France; anita.michel@efs.sante.fr (A.E.); fabienne.proamer@efs.sante.fr (F.P.); 4 Institute for Advanced Biosciences, University Grenoble Alpes, CNRS UMR 5309, INSERM U1209, F-38700 Grenoble, France; anne-sophie.ribba@univ-grenoble-alpes.fr (A.-S.R.); sandrine.fraboulet@univ-grenoble-alpes.fr (S.F.); laurence.lafanechere@univ-grenoble-alpes.fr (L.L.); 5 National Medical Research Centre of Pediatric Hematology, Oncology and Immunology Named after Dmitry Rogachev, 1 Samory Mashela St, 117198 Moscow, Russia; 6 Surgical Research Unit, Faculty of Science and Medicine, University of Fribourg, CH-1700 Fribourg, Switzerland; carmen.gonelle@unifr.ch

**Keywords:** platelet, hemostasis, thrombosis, clot retraction, fibrin, computational modeling

## Abstract

Primary hemostasis consists in the activation of platelets, which spread on the exposed extracellular matrix at the injured vessel surface. Secondary hemostasis, the coagulation cascade, generates a fibrin clot in which activated platelets and other blood cells get trapped. Active platelet-dependent clot retraction reduces the clot volume by extruding the serum. Thus, the clot architecture changes with time of contraction, which may have an important impact on the healing process and the dissolution of the clot, but the precise physiological role of clot retraction is still not completely understood. Since platelets are the only actors to develop force for the retraction of the clot, their distribution within the clot should influence the final clot architecture. We analyzed platelet distributions in intracoronary thrombi and observed that platelets and fibrin co-accumulate in the periphery of retracting clots in vivo. A computational mechanical model suggests that asymmetric forces are responsible for a different contractile behavior of platelets in the periphery versus the clot center, which in turn leads to an uneven distribution of platelets and fibrin fibers within the clot. We developed an in vitro clot retraction assay that reproduces the in vivo observations and follows the prediction of the computational model. Our findings suggest a new active role of platelet contraction in forming a tight fibrin- and platelet-rich boundary layer on the free surface of fibrin clots.

## 1. Introduction

Platelets continuously inspect blood vessel surfaces for damage. Detection of a lesion leads to their activation and spreading on the subendothelial lining. Parallel to the early accumulation of platelets at the injured site, plasma coagulation is initiated, leading to thrombin-mediated cleavage of fibrinogen, which polymerizes into a fibrin network in which platelets and other blood cells get caught. Activated platelets within the fibrin network extend filopodia, and the integrin receptor αIIbβ3 on the platelet surface binds to the fibrin fibers. The subsequent actin/myosin-IIa-dependent contraction of the platelet cytoskeleton [1] and several series of new filopodia extensions followed by their retraction will pull the fibrin fibers together [2]. This leads to the contraction of the clot. The clot retraction process can last for several hours within the circulation [3], and the complete aspects of its physiological role are not yet fully understood. It has been suggested that the role of this process is to tear together the ruptured vessel walls and to reduce clot height to re-establish unhindered blood flow [4]. Indeed, platelets within a clot are able to detect when blood flow ceases and immediately respond by increasing the retraction of the clot [5]. The degree of clot retraction in turn appears to influence clot resorption [6]. In addition to these general physiological aspects, it is very likely that the overall architecture of the clot established during its retraction may have a physiological role.

Several studies have addressed the early phases of clot formation using either in vitro or in vivo assays under flow [7,8]. It has been shown that platelets accumulate at the injury side to form an initial platelet-dense hemostatic plug with a more loosely packed outer platelet shell [8]. Subsequent thrombin-induced conversion of fibrinogen to fibrin leads to the formation of the fibrin network and the stabilization of the initial plug. The organization and distribution of platelets at later stages in the retracted clot has been less well documented. Here, we analyzed pathological thrombi for the distribution of platelets and observed an accumulation of platelets and fibrin in the periphery of these clots. To understand the biophysical rules underlying this observation, we developed a computer model and an in vitro clot retraction assay to simulate and reproduce this phenomenon in vitro. At later stages after clot formation, clots may be more isolated from the bloodstream. We therefore assayed clot retraction under static conditions in order to create experimental settings similar to in vivo conditions. More importantly, this study focuses on the contraction of fibrin clots rather than platelet aggregates, and these clots generally form in vivo in regions of low flow, recirculation, or stagnation. Additionally, clot retraction under different assay conditions can provide complementary information, and some fundamental biophysical properties of clot retraction may become apparent only under static conditions. Clot architecture was analyzed by immunofluorescence after z-projections of confocal image stacks and by transmission electron microscopy.

## 2. Materials and Methods

### 2.1. Reagents

The following reagents were used: neutral formalin (Sigma-Aldrich, Saint-Quentin Fallavier, France), thrombin (Sigma-Aldrich), Pluronic F-127 (Sigma-Aldrich), Mowiol 4-88 (Calbiochem, Paris, France), and mouse anti-integrin αIIb (Santa-Cruz, Heidelberg, Germany, sc-7310).

### 2.2. Preparation of Human Platelet-Rich Plasma (PRP)

Buffy coats from a French blood bank were diluted with an equal volume of phosphate buffered saline (PBS), centrifuged for 10 min, 400× *g*, room temperature (RT), and the upper phase corresponding to the platelet-rich plasma (PRP) was collected. PRP is prepared from buffy coats, usually 2 days after donation (priority is given to patient transfusions). Platelets’ functionality is routinely tested by a spreading assay on a glass surface as previously described [9]. Their capacity to activate and spread on a glass surface ensures that they are fully functional and able to retract a clot.

### 2.3. Clot Retraction Assays

Clots either were allowed to retract unconstrained (Supplementary Figure S1A) or were formed around two holders (two sterile, round, plastic inoculation loops; Dutscher, Bernolsheim, France, ref. 042861), which allows clot contraction under isometric tension and prevents clot collapse (Supplementary Figure S1B). Clots are formed in tubes coated for at least 30 min with a 2% solution of the surfactant Pluronic (to prevent clot adhesion to the tube) and washed once with PBS. Platelet concentration is adjusted to 1 × 10^8^/mL with PPP (platelet-poor plasma), and a small amount of erythrocytes is added for color contrast. At this concentration, platelets are not in contact with each other as estimated by the following calculation. Clots are formed using 400 μL of PRP at 1 × 10^8^ platelets/mL; this means 4 × 10^7^ platelets in an initial volume of 400 μL. The mean platelet volume = 10 fL. The total combined platelet volume of 4 × 10^7^ platelets = 0.4 μL. Thus, the total platelet volume is 1000× smaller than the initial clot volume of 400 μL. The retracted clot between the two holders has an approximate “cylindrical” volume of 100 μL (diameter of holder, 6 mm; clot length, 4 mm). Thus, the total platelet volume is 250× smaller than the retracted clot volume.

Clot formation is initiated by the addition of thrombin to a final concentration of 2.5 U/mL. Clots are fixed for 1 h at RT after different retraction times (as indicated) by the addition of 1 mL isotonic formalin. Thus, clots are not manipulated before fixation, which prevents artefactual squeezing of the clot. Clots are then washed in PBS and incubated in PBS/15% sucrose overnight at 4 °C. After a further incubation step of 4 h at 37 °C in PBS/15% sucrose/7.5% gelatin, clots are embedded in the same gelatin solution and snap-frozen for 1 min in isopentane cooled to −65 °C. Clots are kept at −80 °C until used for cryosectioning.

For fluorescent fibrin clots, 1.2 μL Alexa 488-labeled fibrinogen (10.46 mg/mL) is added to 1 mL PRP containing 1 × 10^8^ platelets.

### 2.4. Immunofluorescence

Using a cryomicrotome, clot sections of 14 μm parallel to the inoculation loops were prepared. Clot sections were permeabilized with PBS/0.2% Triton X-100 for 45 min at RT and then incubated with blocking buffer (3% bovine serum albumin and 10% goat serum in PBS) for 1 h at RT. Sections were then incubated for 2 h with a mouse monoclonal antibody against the integrin αIIb subunit diluted in blocking buffer and then washed twice with PBS and once with PBS/0.2% Triton X-100 and incubated with a secondary antibody against mouse IgG (coupled to Alexa 546; Invitrogen, Illkirch, France) diluted in blocking buffer for 2 h at RT. After three washing steps, coverslips were mounted using Mowiol.

### 2.5. Transmission Electron Microscopy

Clots formed around two holders as described above were fixed after 1 h of retraction by the addition of 1 mL fixative solution, previously warmed to 37 °C, and composed of 2.5% glutaraldehyde in 0.1 M sodium cacodylate buffer containing 2% sucrose (305 mOsm, pH 7.3). The samples were rinsed and postfixed for 1 h at 4 °C with 1% osmium tetroxide in 0.1 M sodium cacodylate buffer. After additional washing in 0.1 M sodium cacodylate buffer, they were dehydrated in successively increasing ethanol concentrations before embedding in epon. Polymerization of the resin was continued at 50 °C for 2 days. Ultrathin sections (100 nm), stained with lead citrate and uranyl acetate, were examined under a Jeol JEM-2100Plus LaB6 electron microscope (120 kV).

### 2.6. Histological Characterization of Human Coronary Thrombi

Thrombi from 84 patients who underwent primary percutaneous coronary intervention were harvested from the occluded coronary artery segments. Recanalization was performed with 0.014″ guide wires, followed by thrombectomy with Export Advance, Eliminate. Retrieved material was fixed with paraformaldehyde, embedded in paraffin, and sectioned, and sections were processed for immunohistochemistry to detect platelets using an antibody against CD42b. The images were acquired with a bright-field light microscope, Nikon Eclipse Ni (Nikon, Paris, France), captured using a Nikon Digital Sight DS-Ri 1 camera and the option grab large images of the Nikon NIS-Elements software (Nikon). The platelet density was measured using the Bersoft Image Measurement software.

### 2.7. Computer Model of Clot Contraction

A mathematical model was designed to describe the contraction of a “one-dimensional” thrombus (a line of platelets). Platelets were modeled as stiff spheres (radius *R* = 1.3 μm) [10], located uniformly within the thrombus at the initial time point and connected with extended elastic springs [2] (Figure 2A). These springs represent extended platelet filopodia attached to fibrin fibers (see Appendix A), and contraction occurs when filopodia retract. Therefore, the equilibrium length of these springs was assumed to be equal to 0 (fully retracted filopodia). The clot is contracted due to the retraction of the springs, and each platelet attracts adjacent platelets according to Hooke’s law *F = k × (*∆*x − l*_0_*)*, where *F* is the force exerted on a platelet by the adjacent one, *k* is the spring coefficient, ∆*x* is the distance between adjacent platelets, and *l*_0_ is the equilibrium length of the spring between platelets (*l*_0_ is equal to 2R). Platelets move in a fibrin gel, which is a viscoelastic environment. The viscosity was added according to Newton’s law *F = −*6 *× π × µ × R × V*, where *µ* is the viscosity of the medium (*µ* = 1 or 10 mPa·s for the fibrin network) [11,12] and *V* is the platelet velocity. The elastic component of the fibrin gel was represented by an additional spring between each pair of platelets. The equilibrium length of this spring is equal to the initial distance between platelets ∆*x*_0_, and this situation corresponds to the undeformed fibrin gel. When platelets get closer to each other, the gel is deformed, and the spring becomes compressed and resists deformation with the force *F_fib_*, which might be calculated according to Hooke’s law *F_fib_ = k_fib_ × (*∆*x*_0_ − ∆*x*), where *F_fib_* is the force exerted on a platelet by the compressed fibrin gel, *k_fib_* is the spring coefficient, ∆*x* is the distance between adjacent platelets, and ∆*x*_0_ is the initial distance between adjacent platelets.

Model equations are generated automatically for N platelets. The initial distance ∆*x*_0_ between platelets is calculated from the platelet concentration with the assumption that platelets are distributed homogeneously at the initial time point. For detailed model descriptions and examples of model equations, see Appendix A.

The two-dimensional model of a clot between two holders was designed to simulate the stationary distribution of platelets after contraction. For model description, see Appendix B.

## 3. Results

### 3.1. Platelet Distribution in Human Intracoronary Thrombi

**Figure 1:**
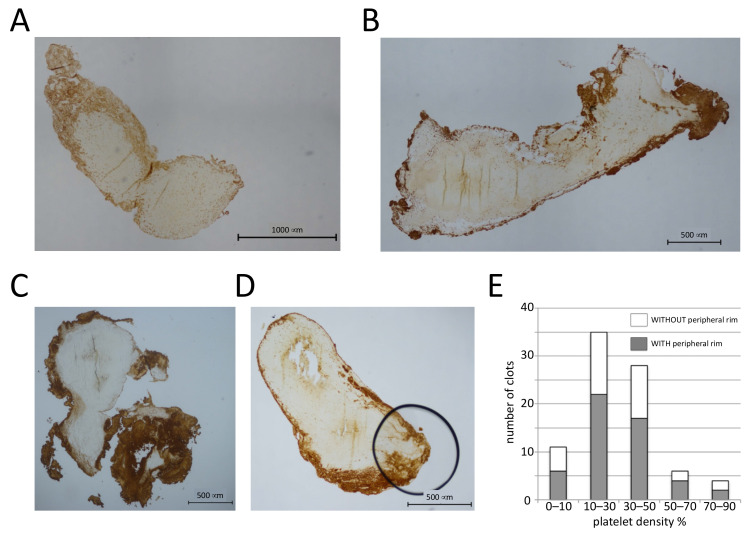
Platelets accumulate in the periphery of human coronary thrombi. (**A**–**D**) Sections (4 μm) of human coronary thrombi stained with an antibody against CD42b. Four different examples of thrombi with a platelet-rich periphery are shown. (**E**) The surface area occupied by platelets was determined, and clots were classified into five different categories based on the platelet density. The number of clots with and without platelet accumulation in the periphery for each category is shown; *n* = 84 clots.

We first investigated platelet distributions in pathological thrombi. We analyzed human arterial thrombi, which can have variable fibrin contents [13,14], often high platelet contents [15], and low erythrocyte numbers. Coronary artery thrombi, recovered from patients during primary percutaneous coronary intervention, were fixed and sectioned, and the platelet distribution was evaluated after immunohistochemical staining for the von Willebrand factor receptor subunit CD42b expressed on the platelet plasma membrane. In total, we observed that 51 out of 84 thrombi have a peripheral accumulation of platelets (Figure 1A–D), and the peripheral platelet rim is between 10 and 500 μm thick. We also determined the surface area of the section occupied by platelets and classified the clot images into five different categories according to platelet density. Thrombi with peripheral platelet accumulation were present in each category (Figure 1E), suggesting that peripheral platelet accumulation is not dependent on platelet numbers. Of note, the presence of a platelet-rich periphery was also independent of the red blood cell density (not shown).

### 3.2. Biophysical Model of Clot Retraction

**Figure 2:**
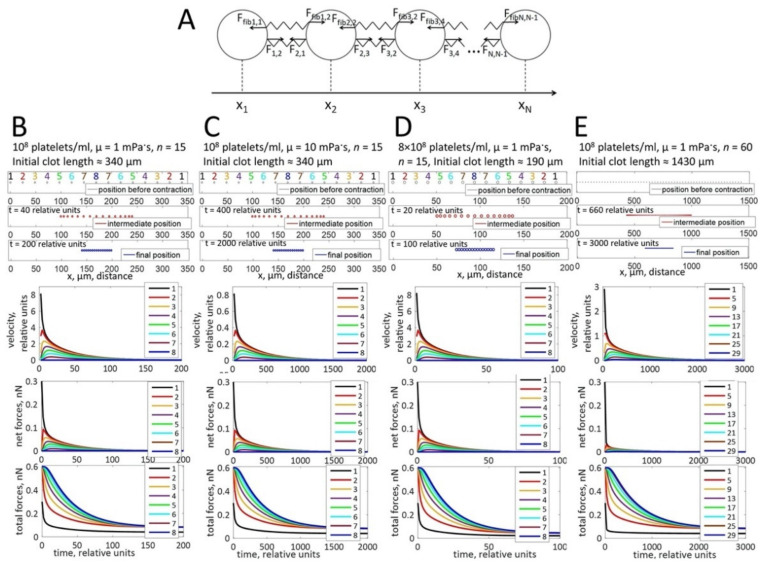
Computational modeling of platelet distributions in a one-dimensional, unconstrained clot. (**A**) Schematic representation of the modeled system. Platelets are depicted as circles, connected by elastic springs. F_i,j_ is the force exerted on a platelet i by a neighboring platelet j pulling on the adjacent fibrin stands. F_fibi,j_ is the force exerted on a platelet i by the compressed fibrin gel. The forces change with the distance between platelets (|x_j_ − x_i_|) according to Hooke’s law. (**B**–**E**) The modeling approach simulates platelet distributions after contraction. Black ovals—initial state of platelet distribution. Blue ovals—platelet distribution after contraction, indicating that the platelets are located uniformly in the retracted clot. Simulations of platelet velocities (upper graphs), net forces (the vector sum of forces that act on a platelet; middle graphs), and total forces (the algebraic sum of all forces that act on a platelet; lower graphs) are shown for different platelet concentrations, different viscosities of the fibrin network (µ), or different clot sizes as indicated at the top of panels B-E. For all conditions, platelets at the border of the thrombus move faster than platelets in the center. The platelet velocity decreases exponentially with time of contraction. Net forces that act on the peripheral platelets are higher than net forces in the center of the clot. In contrast, total forces are higher in the center than in the periphery. Forces are not influenced by the viscosity of the fibrin network and the platelet concentration. Velocities decrease when the viscosity of the fibrin network increases.

To investigate the physical parameters underlying this non-uniform distribution of platelets in these clots, we used a computational modeling approach. Since the fibrin network can be considered a viscoelastic environment [16], we designed a model of clot contraction in a viscoelastic medium to evaluate how the platelet distribution could change upon contraction. The model was based on a one-dimensional chain of interconnected platelets (Figure 2A, Appendix A).

In theory, when all platelets have the same capacity to contract and are homogenously distributed in an infinite clot, then no clot retraction is possible since all platelets counteract each other. However, clots have an edge and platelets are affected by two types of forces: the forces resulting from contracting platelets connected to each other through the fibrin fibers (pulling them together) and an opposing viscous friction force generated by the viscosity of the fibrin network (resisting the pulling forces). According to the supposed force distributions (Figure 2B–E, second graphs), the platelets at the border are subject to a stronger resultant net pulling force than those in the center (because the platelet–fibrin–platelet connections exist for them from one side only, while the contraction forces in the center are balanced). As a result, the computational model predicts that these end-chain platelets move more rapidly (Figure 2B–E, upper graphs) and become closer to the next platelet in the line. This next platelet in turn begins to move more rapidly (because the force pulling it to the middle becomes less balanced). As a result, the clot retracts to the direction of its center. Initially, the gaps between platelets are large in the center of the thrombus, and the distance between peripheral platelets is reduced. However, the final platelet distribution is uniform in the tightly retracted clot (Figure 2B–E, blue ovals).

### 3.3. Platelet Distribution in Clots Formed In Vitro

**Figure 3:**
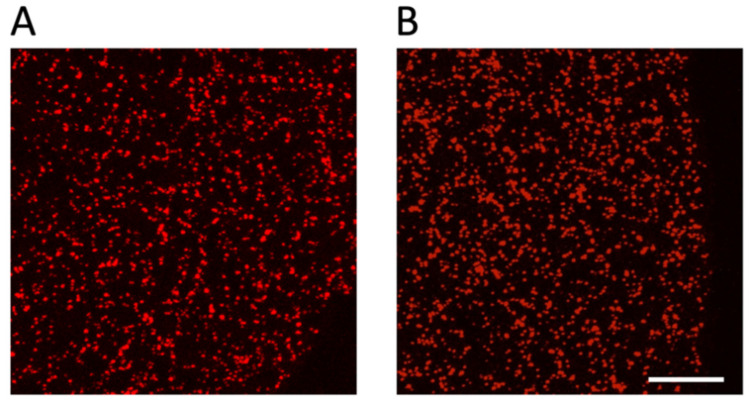
Platelets are homogenously distributed in an unconstrained retracting clot. Platelet-rich plasma (PRP) was either recalcified (**A**) or not (**B**) before induction of clot formation by the addition of thrombin (final concentration of 2.5 U/mL). Clots were fixed after 60 min of retraction at RT, and 14 μm sections were stained to detect the position of platelets using an antibody against αIIb integrins present on the platelet surface; scale bar, 100 μm.

To test the described model, we performed an in vitro clot retraction assay. Although it has been shown previously that clots formed with non-recalcified, citrated PRP retract normally [17], we first tested clot retraction with or without recalcification. No difference was observed between clots recalcified or not and allowed to retract for 60 min at room temperature before fixation. Under both conditions, clots lose about 60% of their initial volume within the same time period (see Supplementary Figure S1). Cryosections of these clots were stained for the integrin subunit αIIb to determine the platelet distribution within the clots. The distribution of platelets in such retracting clots does not show a significant accumulation in the periphery of the clot as shown in Figure 3. Clots allowed to retract for 20 min at 37 °C showed a similar degree of retraction, and platelets were also homogenously distributed (not shown). This result suggests that an important aspect is missing in our modeling approach.

### 3.4. Two-Dimensional Model of Clot Contraction

**Figure 4:**
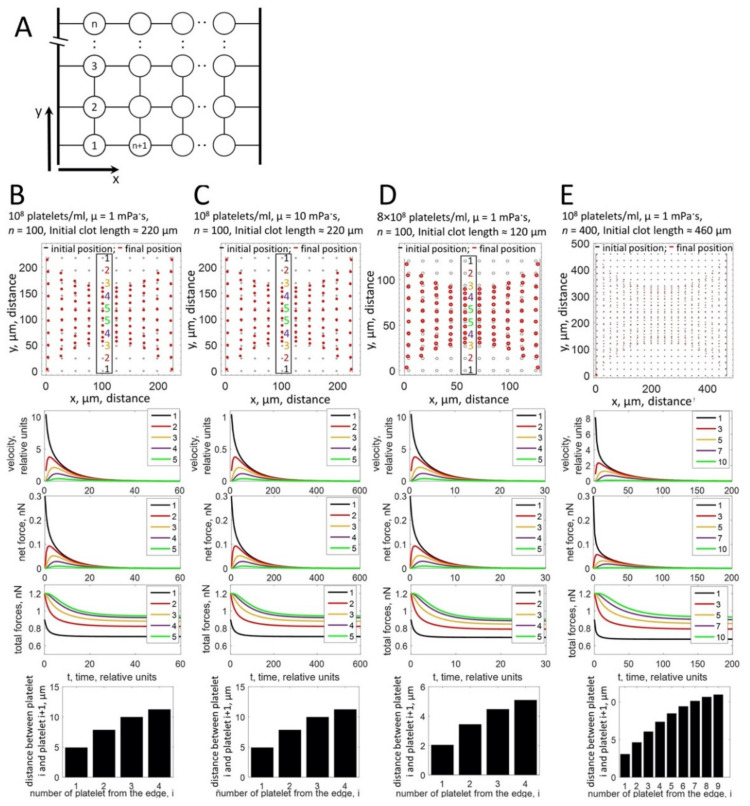
Computational modeling of platelet distributions in a two-dimensional, constrained clot. (**A**) Schematic representation of the modeled system. Platelets are depicted as circles, connected by elastic springs. Holders are modeled as stiff rods. The connection between a platelet and a holder is modeled as an elastic spring. (**B**–**E**) Upper panels: the modeling approach simulates the non-uniform distribution of platelets within the clot after contraction for different platelet concentrations, different viscosities of the fibrin network (µ), or different clot sizes as indicated on top of the panels (**B**–**E**). Black ovals—initial state of platelet distribution. Red ovals—platelet distribution after contraction. For the central part of the system (numbered platelets in the black box), platelets accumulate at the free periphery of the clot, while the distance between central platelets remains large. Middle panels: simulation of platelet velocities (first graph), net forces (the vector sum of forces that act on a platelet; second graph), and total forces (the algebraic sum of all forces that act on a platelet; third graph). Platelets at the free periphery of the thrombus move faster than platelets in the center. The platelet velocity decreases exponentially with time of contraction. Net forces that act on the peripheral platelets are higher than net forces in the center of the clot. For peripheral platelets, net forces are directed towards the center of the clot. For central platelets, forces acting in opposite directions are balanced and net force is close to zero. In contrast, total forces are higher in the center than in the periphery. Forces are not influenced by the viscosity (µ) of the fibrin network and the platelet concentration. Velocities decrease when the viscosity of the fibrin network increases. Lower graphs: the calculation of distances between pairs of platelets in the central column (platelets in the black box in upper panels of (**B**–**E**)) shows that the distance between platelets at the border (bar 1) is significantly smaller than the distances between platelets in the center (bar 4 or 9).

In fact, the fundamental difference between the in vivo and in vitro observations might be that under our in vitro conditions, clots have to be fixed before a steady state can be reached, while in vivo thrombi might be attached to ruptured vessel walls in case of an injury or to atherosclerotic lesions [18] and retract for a longer time period. We thus introduced in our model two holders simulating attachment sites (Appendix B). Under these conditions, the simulations show an accumulation of platelets in the clot periphery when a steady-state contraction is reached (Figure 4B–E, upper panels, red ovals, Supplementary Video S1), which is possible since the tension developed between the holders prevents complete collapse of the clot.

### 3.5. Platelet Distribution in Clots Retracting under Isometric Tension In Vitro

**Figure 5:**
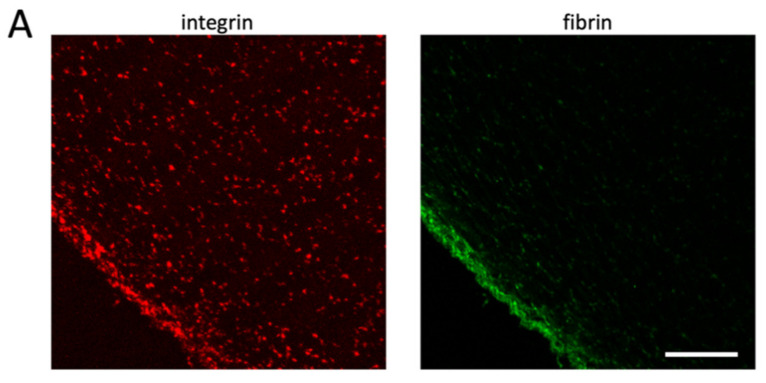

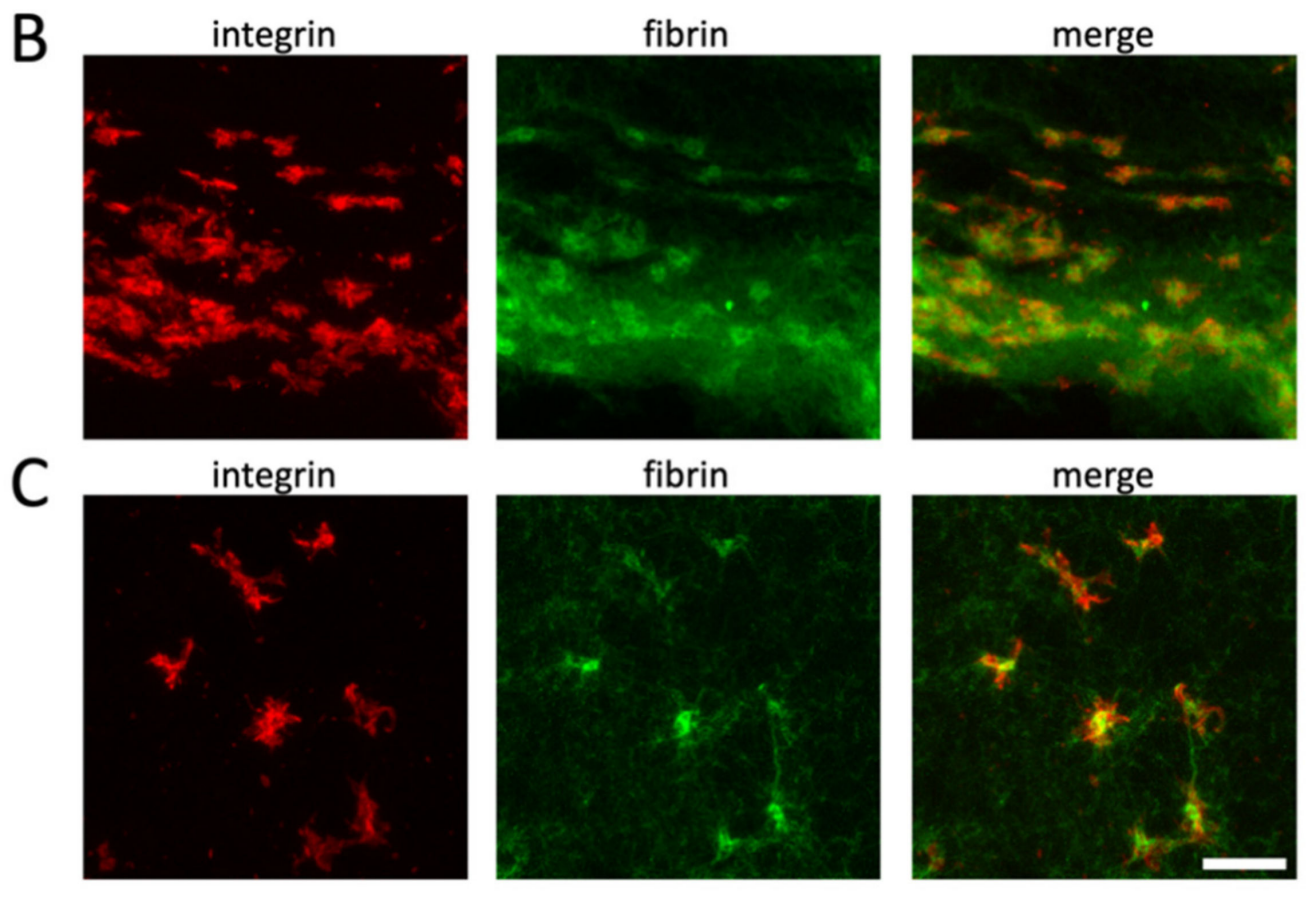
Platelets accumulate in the clot periphery after clot contraction under isometric tension. Alexa 488-labeled fibrinogen was added to PRP before the induction of clot formation in tubes containing two holders (see Supplementary Figure S1B). Clots were fixed after 3 h of retraction at 37 °C, and sections were stained for αIIb integrins. Left image shows integrin staining (red), and right image shows fibrin fibers (green); scale bar, 100 μm (**A**). Higher-resolution images show that platelets are more compact in the clot periphery (**B**) than in the clot center (**C**); scale bar, 10 μm.

To verify the model predictions, we decided to establish experimental conditions that mimic the model and the physiological situation, where clots adhere to different sites of a vessel. We, thus, developed a clot retraction assay where clots are formed around two holders. Similar to in vivo conditions, these clots retract until a steady state is reached, and in contrast to unconstrained clot retraction, “clot collapse” is prevented even during prolonged incubation times (Supplementary Figure S1B). Since no difference has been observed between no-holder clots with and without recalcification (Figure 3 and Supplementary Figure S1A), we used in the following only clots formed with non-recalcified PRP. The number of platelets was adjusted so that in principle, two neighboring platelets are not in contact with each other in the retracted clot and therefore attach only to fibrin fibers (1 × 10^8^/mL; see the Materials and Methods section for calculation). The middle of the clot was cut in a transversal orientation to obtain sections with a free clot periphery (far away from the holders; Supplementary Figure S1B). These sections were stained for the integrin receptor to reveal the position of platelets. Under these conditions, the homogenous distribution of platelets is not seen; instead, platelets accumulate in the periphery in accordance with the model predictions (Figure 5A, left panel). Fibrin fibers are associated with platelets and should also accumulate in the periphery of the retracting clot, since platelets pull on the fibrin fibers and compact them. Fiber compaction should be more efficient in the periphery than in the clot center, where the resisting forces are higher. To investigate that, we spiked PRP with Alexa 488-labeled fibrinogen before the induction of clot formation. As expected, fibrin fibers also concentrate in the periphery of the contracting clot together with the platelets (Figure 5A, right panel).

The model also predicts that platelets located in the center of the clot stay in a three-dimensional elongated state due to the balance of pulling forces from opposite directions (Figure 4B–E, lower panels). They are therefore unable to fully contract, resulting in the lower density of the central fibrin fibers observed in Figure 5A. To confirm this hypothesis, we decided to compare the morphological aspect of platelets present in the border and center regions of the clot. To this end, we acquired confocal image stacks at a higher magnification to produce z-stack projections of clot sections. It is often not possible to distinguish individual platelets in the clot periphery. However, those that can be distinguished appear to have a more compact morphology compared with platelets in the center, which are still “spread” within the fibrin network with several extended filopodia (Figure 5B,C, respectively).

The simulations shown in Figure 4 were obtained for clots that were significantly smaller than the clots formed in the in vitro assay (100 or 400 platelets in simulations in Figure 4 versus 4 × 10^7^ platelets in clots formed in vitro). To validate our modeling approach, a two-dimensional model of the real-size clot was developed (Appendix C), which produced a timescale of contraction comparable (Supplementary Figure S2) to the experimentally observed time needed for complete retraction.

**Figure 6:**
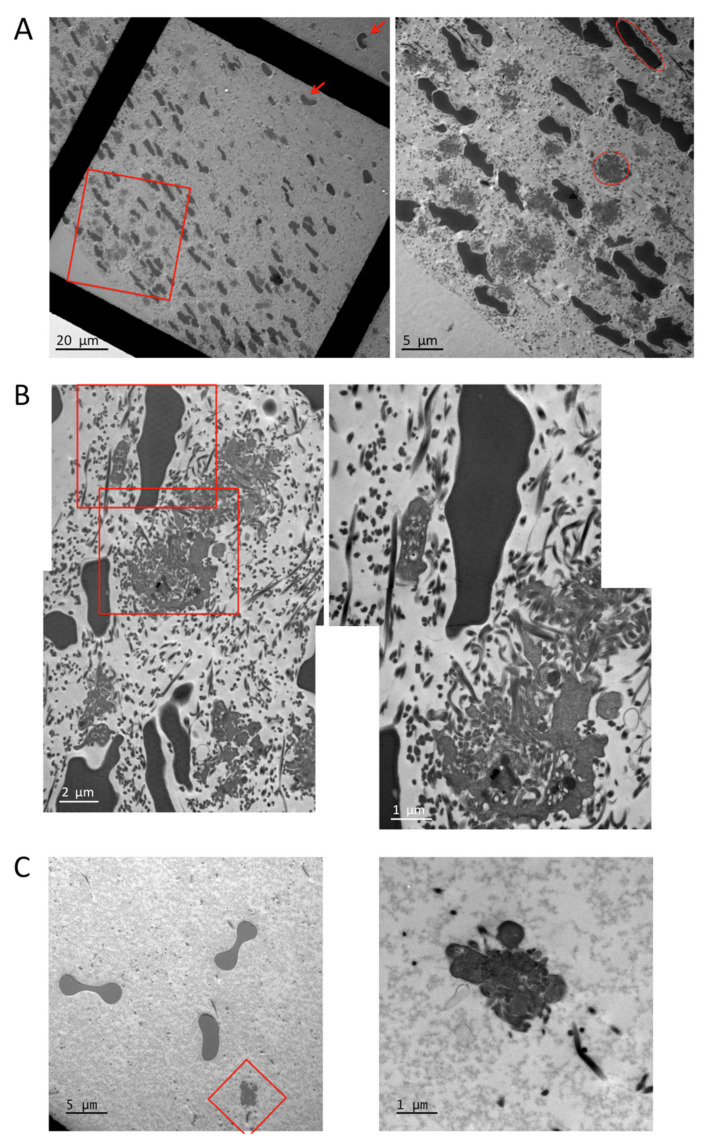
Transmission electron microscopy of clot sections. (**A**) Overview of clot sections, left panel (red arrows indicate undeformed erythrocytes closer to the clot center), and enlargement of the clot periphery as indicated by the red rectangle, right panel (red circle indicates a typical platelet cut approximately in its center; red ellipse indicates a typical compressed erythrocyte cut approximately in its center). (**B**) Enlargement of periphery, left panel, and higher magnification of the same image as indicated by the red rectangles. (**C**) Enlargement of the clot center, left panel, and higher magnification of the same image as indicated by the red square.

To investigate the clot architecture and platelet morphology in more detail, we performed transmission electron microscopy of transversal clot sections (Figure 6). Platelets, erythrocytes (added for color contrast; see Materials and Methods section), and fibrin fibers are highly concentrated in the clot periphery and sparse in the clot center. The erythrocytes present in the periphery are compressed and oriented, while they have a normal morphology and a random orientation when they are closer to the clot center (arrows, left panel of Figure 6A,C). Fibrin fibers are mainly cut in a transversal orientation both in the periphery and in the center of the clot, indicating that fibers orient along the direction of the tension developed between the two holders. However, fibers are often curved when in close proximity to platelets (Figure 6B). In the periphery, sections of platelet pseudopodia can be observed as well as sections of individual platelets (red circle, right panel, Figure 6A). A higher magnification suggests that peripheral platelets collect fibrin fibers by wrapping their pseudopods around the fibers before retracting them. This leads to an intermingled organization of platelet extensions and curved fibrin fibers. The sections of these retracted platelet/fibrin assemblies are round and have a variable diameter depending at which level they have been sectioned and suggesting that they have a spherical or ellipsoid form. The largest diameters were measured and were found to be between 2.3 and 6.6 µm (mean 4.43 µm, *n* = 34). This can be considered the diameter of a retracted platelet in the periphery (only sections with clearly distinguishable platelet elements have been taken into account; smaller sections of pseudopods have been avoided).

In the clot center, fibrin fibers, erythrocytes, and platelets are sparse, and only sections of platelet pseudopodia with some adjacent fibrin fibers are visible (Figure 6C). These observations confirm the immunofluorescence results and the predictions of the clot retraction model, which show and predict, respectively, that platelets and fibrin fibers are dense in the periphery and distant in the clot center.

## 4. Discussion

The main observation of the present study is that blood clots that undergo retraction under moderate isometric tension end up in a spatially heterogeneous state with platelets and fibrin fibers enriched in the clot periphery. Computer simulations of the contraction mechanics suggest that this phenomenon is due to the asymmetry of contracting forces that arise within the clot. Since this phenomenon occurs also in vivo, it is of physiological and pathological significance. The peripheral platelet accumulation and the fibrin fiber layer may isolate the clot from the blood flow.

The transition from a random distribution of platelets in a fibrin fiber network to a structured clot architecture can be considered an example of a self-organizing living system. Self-organization is observed during developmental processes, leading to tissue differentiation, growth, and renewal. Usually, self-organizing cellular systems rely on a coordinated behavior of cells that adapt gene expression, proliferation, movement, and signaling to their microenvironment. It has been suggested that intercellular communications are based on feedback regulations influencing the behavior of neighboring cells as a function of polarity, mechanical forces, and fate [19]. In the case of blood clots described here, self-organization does not depend on the regulation of gene expression or proliferation. However, the organization of the clot clearly depends on polarity, mechanics, and cell fate. In fact, the polarity is established by the tension developed between the two holders: mechanical forces are generated by the contractile behavior of platelets, and cell fate changes are related to the differential morphologies of platelets and erythrocytes in the periphery versus the clot center, which in turn may influence the polarity and the contraction as a feedback regulatory mechanism.

The difference in morphology between platelets located in the center versus those in the periphery of the clot confirms the prediction of our computational modeling approach. Furthermore, the simulation showing that platelets in the periphery “move” faster than those in the center is in agreement with results published by Kim et al., showing that, indeed, platelets observed at the border of a clot move with a higher speed compared with those in the center [2]. The compression of peripheral but not central erythrocytes also reflects the fact that the forces to which they are exposed are stronger in the periphery than in the center. It has been shown previously that erythrocytes in whole blood clots are compacted to form polyhedrocytes [4]. The compressed erythrocytes observed here may be considered pre-polyhedral cells, and it is interesting to note that they do not have to be densely packed to undergo these morphological shape changes. Fibrin fiber accumulation in the periphery of the clot is another consequence of the asymmetric contractile behavior of the platelets. The combined buildup of fibrin fibers and platelets could reinforce the clot boundary by strengthening the fibrin biofilm layer, which has been shown to cover clot surfaces [20]. Such a fibrin biofilm has also been observed on intracoronary thrombi of patients with acute myocardial infarction [21]. The fibrin biofilm, in conjunction with the accumulated platelets and fibrin fibers, may help seal the clot to avoid agonist washout, block the adhesion of other circulating platelets, regulate fibrinolysis, and prevent bacterial invasion of the clot in the event of an external injury.

After injury, platelets accumulate within seconds at the wound site, forming an initial plug in the absence of thrombin and fibrin fibers [8,22]. Platelets are more densely packed in the core of this initial plug and less in the periphery. This is in agreement with our model predictions since only platelets getting trapped at later time points in the forming fibrin network should accumulate in the periphery. Our results are in accordance with earlier studies analyzing the structure of intracoronary thrombi of patients, which have shown that higher fibrin and platelet contents were found on the clot surface when compared with the inner part of the clot [14,23]. Furthermore, other types of pathological thrombi, such as cerebral or venous thrombi and pulmonary emboli, show a peripheral accumulation of platelets and fibrin fibers [23,24,25]. The fact that the majority of in vivo-formed thrombi show this peripheral platelet rim is rather surprising because one can expect a high heterogeneity between different clots in terms of local platelet and fibrin concentrations. In this respect, it is interesting to note that within the coronary thrombi examined here, there is a high variability in terms of platelet density. However, clots with a platelet-rich periphery are present in all density categories. This is in line with our simulations, which show that platelets accumulate at the periphery regardless of their initial density. Our model further predicts that platelets will also accumulate in the clot periphery in more viscous clots with higher fibrin concentrations. A study by Lam et al. has shown that platelets can adjust their contractile force according to the local stiffness of their environment [26]. Together, this may explain why a peripheral accumulation of platelets can also be observed in quite irregular clots with different regions of rigidity and platelet concentrations. Furthermore, it has been observed that even in the presence of high numbers of red blood cells, platelets and fibrin fibers accumulate at the periphery of the clots [4], suggesting that this phenomenon is important for all sorts of thrombi in order to strengthen them and isolate them from the surrounding blood flow.

Previous studies have suggested that contraction may play a role in changing the architecture of platelet-rich aggregates by expelling procoagulant platelets to the periphery, leading to more fibrin formed in the peripheral region of the clot [27]. Here, we investigate an opposite process. A clot formed mostly by fibrin is contracted by platelets so that platelets and associated fibrin fibers are enriched in the periphery. It is interesting that these rather different mechanisms lead to the same outcome, that is, increased fibrin density at the periphery of the thrombus, whether it is a “white” platelet thrombus or a “red” fibrin-rich clot. This may suggest a general significance of the fibrin-rich layers formed by contraction.

The characterization of the structure of thrombi that we conducted and our effort to understand the cellular and biophysical mechanisms underlying the organization of the clot structure is a prerequisite for improvements of therapeutic interventions following acute infarction or stroke. It may also help to find strategies to improve and accelerate the healing process of internal or external injuries.

## Data Availability

All data are available in the article or supplementary material.

## Supplementary Materials

The following are available online at https://www.mdpi.com/2073-4409/10/3/584/s1: Figure S1. Illustration of clot retraction assays. (**A**) Clots were induced using non-recalcified or recalcified PRP (10 mM final CaCl_2_ concentration) and fixed after 1 h of retraction at RT. (**B**) Elaboration of a clot retraction assay around two holders. The strong clot adhesion to both holders allows clot retraction but prevents “clot collapse” even after prolonged incubation times (here, 3 h at 37 °C). Figure S2. Computational modeling of platelet distributions in a real-size one- and two-dimensional clot. (**A**) Schematic representation of the modeled system of N platelets. Platelets are depicted as circles connected by elastic springs. The system was divided into two subsystems, each of them consisting of N/2 platelets. (**B**,**C**) Schematic representation of the real-size 1D (**B**) and 2D (**C**) systems. The subsystems of platelets are depicted as squares connected by elastic springs with the spring coefficient ksub. The 2D system is simulated without holders. (**D**,**E**) The calculated velocities and distances between subsystems of platelets in 1D (**D**) and 2D (**E**) real-size models. The number of subsystems is 34 for 1D and 34 × 34 for 2D clots. For the 2D model, the results are shown for the central row of subsystems. Platelet concentration is 10^8^ platelets/mL; viscosity is μ = 10 mPa·s. Subsystems at the free periphery of the thrombus (data 1) move faster than subsystems in the center (data 17). The platelet velocity decreases exponentially with time of contraction. The contraction is finished in 15 min. Video S1. Platelet movements during clot retraction simulated by the two-dimensional clot model.

## Appendix A. One-Dimensional Mathematical Model

Model equations were designed following Newton’s laws of motion for platelets with the assumption that platelets move without acceleration. For example, the equation for the second platelet in a row was as follows:

$$\begin{array}{c} m\frac{d^{2}x_{2}}{dt^{2}}=-6\cdot \pi \cdot \mu \cdot R\cdot \frac{dx_{2}}{dt}-k\left(x_{2}-x_{1}-2R-l_{0}\right)+k\left(x_{3}-x_{2}-2R-l_{0}\right)\;+\;k_{fib}\\ \cdot \left(\Delta x_{0}-\left(x_{2}-x_{1}\right)\right)-k_{fib}\cdot \left(\Delta x_{0}-\left(x_{3}-x_{2}\right)\right)=0 \end{array}$$

*l*_0_ = 0 is the equilibrium length of the spring, which attracts platelets to each other; *m* is the platelet mass; and *x_i_* is the coordinate of the platelet *i* center.

Initial distance ∆*x*_0_ between platelets was calculated from the platelet concentration with the assumption that platelets were distributed homogeneously at the initial time point. For example, the platelet concentration was 1 × 10^8^ platelets/mL or 1 × 10^8^ platelets/cm^3^, and the distribution of platelets was homogeneous. Therefore, there was a line of (1 × 10^8^)^1/3^ = 464 platelets along 1 cm. Thus, the distance between two adjacent platelets was equal to 1 cm/464 = 21.6 µm.

The spring coefficient *k* was calculated from the following considerations:

(1) The mean length of the platelet filopodia is equal to 2.7 µm (L_filap_ = 2.7 µm) [2]. The force generated by one filopodium is equal to 0.3 nN [2]. Each filopodium was modeled as a spring with a spring coefficient of k_filap_ = 0.3 nN/L_filap_ = 11.12 × 10^−2^ g/s^2^.
(2) The elementary step of clot contraction is “sequential extension–retraction of platelet filopodia” [2]. Therefore, the elastic spring between adjacent platelets might be modeled as a sequence of springs with a spring coefficient of k_filap_ connected from end to end. The number of these springs between adjacent platelets was calculated as *w* = ∆*x*_0_/L_filap_. Additionally, the overall spring coefficient of the sequence of filopodia was calculated as k = k_filap_/*w*.

The spring coefficient for the fibrin gel is equal to *k_fib_* = (1 × 10^−3^) g/s^2^.

Values of model parameters are listed in the Table A1. Model equations were solved numerically using the Dormand–Prince method (Matlab solver ode45). The program was implemented in Matlab (2015b), MathWorks, Inc., Natick, MA, USA.

**Table A1:** Model parameters used in calculations.

Model Parameter	Value
*k*	(11.12 × 10^−2^/*w*) g/s^2^
*k_fib_*	(1 × 10^−3^) g/s^2^
*w*	8 for 1 × 10^8^ platelets/mL4 for 8 × 10^8^ platelets/mL
*µ*	1 or 10 mPa·s
*R*	1.3 μm

## Appendix B. Two-Dimensional Mathematical Model

The two-dimensional model was designed according to the scheme shown in Figure 4. Platelets were modeled as stiff spheres (radius *R* = 1.3 μm), located uniformly within the thrombus at the initial time point, and connected with extended elastic springs (Figure 4A). There were *n* platelets in a row and *n* platelets in a column, so the number of platelets in the clot was *N* = *n* × *n*. The other parameters were the same as for the 1D model.

Holders were modeled as stiff rods. The initial distance between the first line of platelets and the holder was *h*. The connection between the platelet and the holder was modeled as an elastic spring with equilibrium length *h* and spring coefficient *k*_1_ (*k*_1_ = 1000 *× k*).

Model equations were designed following Newton’s laws of motion for platelets with the assumption that platelets move without acceleration. For example, the equation for the *x* and *y* coordinates of the first platelet was as follows:

$$\begin{array}{c} m\frac{d^{2}x_{1}}{dt^{2}}=-6\cdot \pi \cdot \mu \cdot R\cdot \frac{dx_{1}}{dt}+k\;\cdot \;\left(l_{1,2}-2R-l_{0}\right)\;\cdot \;cos\alpha +k\;\cdot \;\left(l_{1,n+1}-2R-l_{0}\right)\;\cdot \;cos\beta -k_{1}\;\cdot \;(l_{h,1}\\ -R-l_{0})\;\cdot \;cos\gamma -k_{fib}\;\cdot \;\left(l_{init}-l_{1,2}\right)\;\cdot \;cos\alpha -k_{fib}\;\cdot \;\left(l_{init}-l_{1,n+1}\right)\;\cdot \;cos\beta \;=\;0 \end{array}$$

$$\begin{array}{c} m\frac{d^{2}y_{1}}{dt^{2}}=-6\cdot \pi \cdot \mu \cdot R\cdot \frac{dy_{1}}{dt}+k\;\cdot \;\left(l_{1,2}-2R-l_{0}\right)\;\cdot \;sin\alpha +k\;\cdot \;\left(l_{1,n+1}-2R-l_{0}\right)\;\cdot \;sin\beta -k_{1}\;\cdot \;(l_{h,1}\\ -R-l_{0})\;\cdot \;sin\gamma -k_{fib}\;\cdot \;\left(l_{init}-l_{1,2}\right)\;\cdot \;sin\alpha -k_{fib}\;\cdot \;\left(l_{init}-l_{1,n+1}\right)\;\cdot \;sin\beta =0 \end{array}$$

*F_i,j_ = k(l_i,j_ −* 2*R − l*_0_*)* is the attractive force acting between platelets *i* and *j*; *l_i,j_* is the distance between the centers of platelets *i* and *j*; *l*_0_ = 0 is the equilibrium length of the spring, which attracts platelets to each other; *α* is the angle between the axis *x* and the force *F*_1,2_ direction; and β is the angle between the axis *x* and the force *F*_1_*_,n+_*_1_ direction. *F_h_,*_1_ *= k*_1_*(l_h,_*_1_ *− R − l*_0_*)* is the attractive force acting between platelet 1 and the holder; *l_h,_*_1_ is the distance between the center of platelet 1 and the holder; and γ is the angle between the axis *x* and the force *F_h_*_,1_ direction. *F_fibi,j_ = k_fib_(l_init_ − l_i,j_)* is the repulsive force that is exerted on the platelet *i* by the compressed fibrin gel.

Values of model parameters are listed in Table A2.

**Table A2:** Model parameters used in calculations.

Model Parameter	Value
*k*	(11.12 × 10^−2^/*w*) g/s^2^
*k*_1_	1000 × k
*k_fib_*	(1 × 10^−3^) g/s^2^
*h*	2.7 µm
*w*	8 for 1 × 10^8^ platelets/mL4 for 8 × 10^8^ platelets/mL
*µ*	1 or 10 mPa·s
*R*	1.3 μm

## Appendix C. One-Dimensional and Two-Dimensional Mathematical Models of the Real-Size Clot

The clot that was formed in the in vitro assay was 400 μL in volume and contained 4 × 10^7^ platelets. That means that there were (4 × 10^7^)^1/3^ = 340 platelets in a line. To evaluate the time of contraction in the real-size clot and to compare it with the experimental value, the system of 340 or 340 × 340 platelets was simulated using the following approach:

(1) The system of N = 20 platelets was simulated using the one-dimensional model from Appendix A (Figure 2A and Figure S2A). The system was divided into two subsystems, each of them consisting of N/2 = 10 platelets. The attractive force F_N/2,N/2+1_, which was exerted on subsystem 1 by subsystem 2, was calculated at the initial time point (F_N/2,N/2+1_(initial) = 0.3 nN). The initial distance between two subsystems was equal to the initial distance between platelets (∆*x*_0_ = 21.6 µm). The spring coefficient for the spring between two subsystems was equal to *k_sub_* = F_N/2,N/2+1_(initial)/∆*x*_0_. The radius of the subsystem was equal to Rsub = (10 × 2R + 9 × ∆*x*_0_)/2 = 110.2 µm.
(2) Using values from step 1, the individual platelets in 1D or 2D models from Appendixes A and B were substituted by the subsystems of platelets (10 or 10 × 10 platelets in a subsystem) (Figure S2B,C). The two-dimensional model was used without holders (k_1_ = 0). The results for 34 or 34 × 34 subsystems are shown in Figure S2D,E. The retraction is completed in 15 min, which is close to the experimental value. We modeled a 2D real-size clot for computational simplicity, but the phenomenon should not be fundamentally different in a 3D system.
